# Sensorimotor control in the congenital absence of functional muscle spindles

**DOI:** 10.1113/EP090768

**Published:** 2023-04-08

**Authors:** Vaughan G. Macefield, Lyndon J. Smith, Lucy Norcliffe‐Kaufmann, Jose‐Alberto Palma, Horacio Kaufmann

**Affiliations:** ^1^ Department of Neuroscience Monash University Melbourne Victoria Australia; ^2^ School of Medicine Western Sydney University Sydney New South Wales Australia; ^3^ Dysautonomia Center, Department of Neurology New York University School of Medicine New York NY USA

**Keywords:** ataxia, cutaneous afferents, HSAN III, microneurography, muscle spindle afferents

## Abstract

Hereditary sensory and autonomic neuropathy type III (HSAN III), also known as familial dysautonomia or Riley–Day syndrome, results from an autosomal recessive genetic mutation that causes a selective loss of specific sensory neurones, leading to greatly elevated pain and temperature thresholds, poor proprioception, marked ataxia and disturbances in blood pressure control. Stretch reflexes are absent throughout the body, which can be explained by the absence of functional muscle spindle afferents – assessed by intraneural microelectrodes inserted into peripheral nerves in the upper and lower limbs. This also explains the greatly compromised proprioception at the knee joint, as assessed by passive joint‐angle matching. Moreover, there is a tight correlation between loss of proprioceptive acuity at the knee and the severity of gait impairment. Surprisingly, proprioception is normal at the elbow, suggesting that participants are relying more on sensory cues from the overlying skin; microelectrode recordings have shown that myelinated tactile afferents in the upper and lower limbs appear to be normal. Nevertheless, the lack of muscle spindles does affect sensorimotor control in the upper limb: in addition to poor performance in the finger‐to‐nose test, manual performance in the Purdue pegboard task is much worse than in age‐matched healthy controls. Unlike those rare individuals with large‐fibre sensory neuropathy, in which both muscle spindle and cutaneous afferents are absent, those with HSAN III present as a means of assessing sensorimotor control following the selective loss of muscle spindle afferents.

## Abstract (highlights)

- **What is the topic of this review?**
  Hereditary sensory and autonomic neuropathy type III (HSAN III).
- **What advances does it highlight?**
  In individuals with (HSAN III) functional muscle spindles appear to be absent throughout the body, though myelinated cutaneous afferents are present. The former may explain the poor proprioception at the knee joint, while the latter may explain why increasing cutaneous feedback improves proprioception at the knee. Reaching and lifting small objects was greatly compromised, arguing for an important role of muscles spindles in sensorimotor control.

## 1 INTRODUCTION

Hereditary sensory and autonomic neuropathy type III (HSAN type III) results from an autosomal recessive genetic mutation on chromosome 9q that causes a deficiency of IκB kinase complex‐associated protein (IKBKAP), leading to a reduction in expression of elongator complex protein 1 (ELP‐1) (Anderson et al., 2001; Blumenfeld et al., 1993; Slaugenhaupt et al., 2001). Initially described as Riley–Day syndrome, and now more commonly referred to as familial dysautonomia, it is limited to members of the Ashkenazi Jewish population and affects many sensory systems (for review see Axelrod, 2002; Norcliffe‐Kaufmann et al., 2017; Palma et al., 2014). Affected individuals exhibit markedly elevated pain and temperature thresholds, leading to a relative indifference to pain, as well as marked ataxia in the upper and especially the lower limbs, the latter leading to a progressively worsening gait that ultimately requires the adoption of walking aids. What causes the ataxia is unknown, but it is known that the problem does not lie in the muscles as tone and strength are normal; moreover, the ataxia is not due to atrophy in the cerebellum, for which there is little evidence (Axelrod et al., 2010; Cohen & Solomon, 1955). Given that tendon reflexes and H‐reflexes are absent throughout the body (Aguayo et al., 1971; Macefield et al., 2011; Mahloudji et al. 1970; Riley 1974), this suggests that the ataxia may be related to a sensory disturbance.

### 1.1 Functional muscle spindles are absent in HSAN III

**FIGURE 1:**
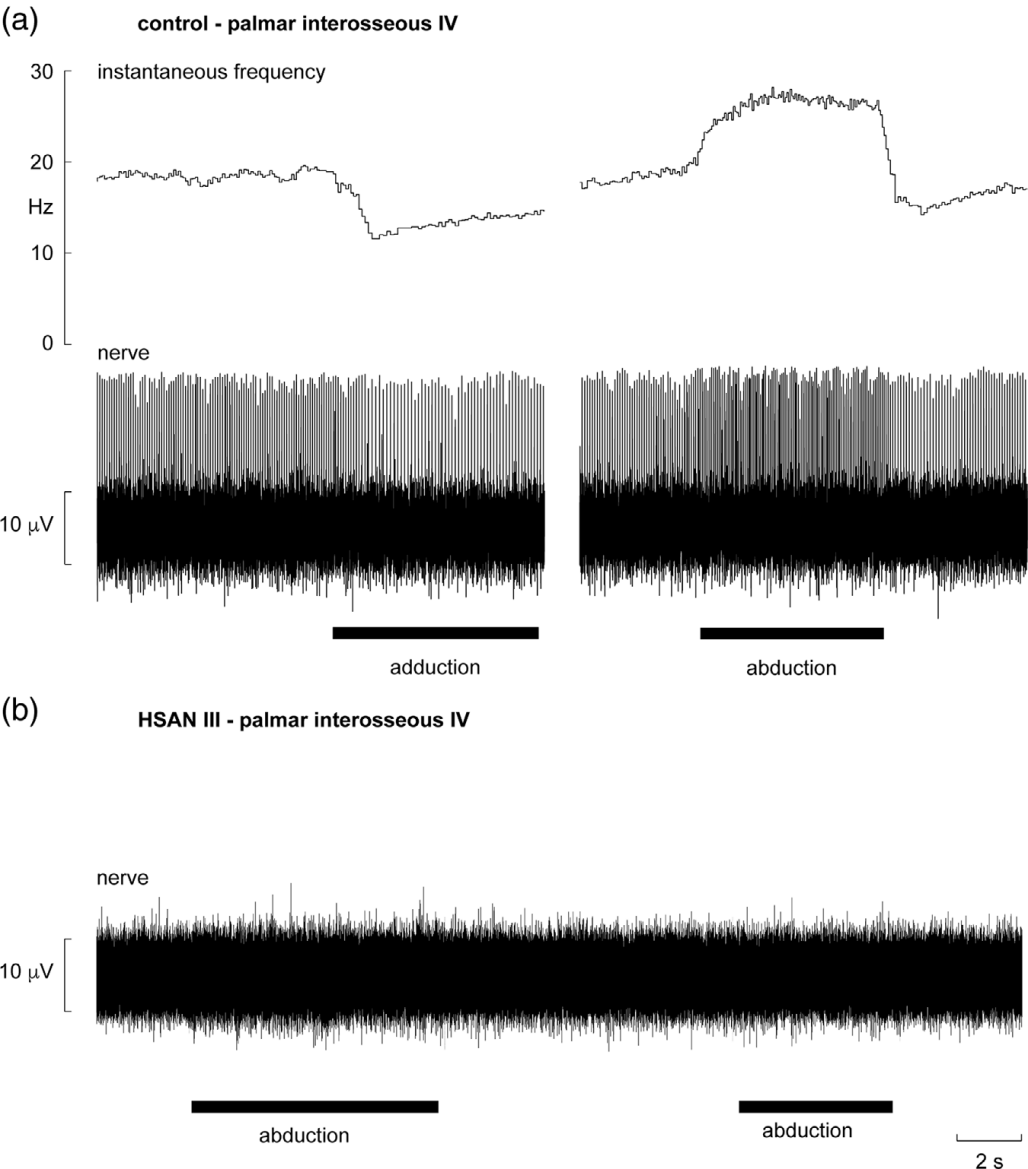
(a) Microelectrode recording from a muscle fascicle of the ulnar nerve, supplying the fourth palmar interosseous muscle, in a healthy control subject. This muscle spindle secondary ending was spontaneously active at rest, and its firing rate could be decreased by passively moving digit V toward digit IV, which unloaded the muscle spindle ending, and increased by passive abduction of digit V, which stretched the receptor‐bearing muscle and hence loaded the muscle spindle. (b) A recording from the same fascicle in a patient with HSAN III failed to reveal any spontaneous or stretch‐evoked afferent activity. Reproduced, with permission, from Smith et al. (2018).

Muscle spindles are highly specialised encapsulated mechanoreceptors, located in skeletal muscles throughout the body, that encode static muscle length as well as being exquisitely sensitive to dynamic muscle stretch; they are unique in being the only mechanoreceptors in the somatosensory system that have their own motor innervation (γ‐motoneurones) that control the sensitivity of the muscle spindle primary and secondary endings (for review see Banks, 2023). While much of this work has been conducted on experimental animals, we have also learnt much from microelectrode recordings from individual muscle spindle afferents in humans (for review see Macefield & Knellwolf, 2018). Because muscle spindles can encode changes in muscle length they can signal changes in joint angle; as such they are our primary proprioceptors, even though mechanoreceptors in the skin (and to a much smaller extent, joint receptors) also contribute (for review see Macefield, 2021). Studies we have performed at the Dysautonomia Center at New York University demonstrated that functional muscle spindle afferents, as recorded via tungsten microelectrodes inserted percutaneously into muscle fascicles of the common peroneal nerve, are absent in individuals with HSAN III (Macefield et al., 2011). We went on to explore whether functional muscle spindles are also absent in the upper limb, choosing to record from muscle fascicles of the ulnar nerve at the wrist so we could assess the properties of muscle spindles in the intrinsic muscles of the hand (Smith et al., 2018). Again, as shown in Figure 1, we were met with a striking silence in participants with HSAN III: there was no spontaneous activity and no stretch‐evoked activity in muscle spindle afferents, in marked contrast to the rich activity one normally finds in neurologically intact individuals. For both nerves we performed an extensive search within several muscle fascicles in each patient. If muscle spindle afferents were present we would have easily found them; after all, they have large‐diameter myelinated axons and you cannot miss them in healthy individuals. Even people with complete spinal cord injury have ongoing activity in muscle spindle afferents originating in muscles below the lesion, despite the clear paralysis of the parent muscles; indeed, the spontaneous firing rates of these de‐efferented muscle spindles are identical to those recorded from intact individuals (Macefield, 2013). Curiously, spontaneous bursts of muscle sympathetic nerve activity (MSNA), which occur with a dominant cardiac rhythmicity, were also absent in HSAN III (Macefield et al., 2013b), contributing to the overall silence one encounters when penetrating muscle fascicles of peripheral nerves in HSAN III. The lack of cardiac rhythmicity of MSNA can be explained by loss of the negative feedback provided by baroreceptor afferents (Norcliffe‐Kaufmann et al., 2010). However, tonic activity of post‐ganglionic sympathetic axons, which increased during emotional stress, was observed (Macefield et al., 2013b), and muscle pain could be evoked by intraneural stimulation within muscle fascicles (Macefield et al., 2011; Smith et al., 2018), suggesting that HSAN III is not associated with loss of either unmyelinated motor (sympathetic) or sensory (pain) axons supplying muscle.

### 1.2 Tactile afferents are present in HSAN III

**FIGURE 2:**
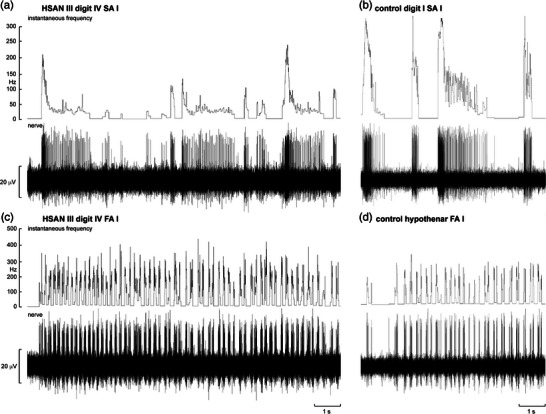
Microelectrode recordings from cutaneous fascicles of the ulnar nerve in a patient with HSAN III (a, c) and a healthy control (b, d). (a) Responses of a slowly adapting type I (SA I) afferent located on the finger pad of digit IV during repeated and sustained indentations with a 1 mm probe of one of the hotspots within its receptive field. Spikes were generated during each indentation. (b) Similar responses from an SA I afferent recorded from digit I in a control. (c) Responses of a fast‐adapting type I (FA I) afferent located on the middle phalanx of digit IV during repeated back‐and‐forth stroking over its receptive field. Spikes were generated during each pass across the receptive field. (d) Similar responses from an FA I afferent recorded from the hypothenar eminence in a control. Reproduced, with permission, from Smith et al. (2018).

Despite the marked silence of recordings obtained from muscle fascicles in HSAN III, when microelectrodes were inserted into cutaneous fascicles of either the common peroneal or ulnar nerves the story was quite different: multi‐unit activity in low‐threshold mechanoreceptor afferents could readily be evoked by stroking over the fascicular innervation zone (Macefield et al., 2011; Smith et al., 2018). Moreover, as shown in Figure 2, single‐unit recordings showed that the physiology of various low‐threshold myelinated tactile afferents was essentially normal, though an extensive quantitative survey was not undertaken as this would require very large samples from all afferent classes (Smith et al., 2018). However, although we have no direct recordings from low‐threshold C‐tactile (CT) afferents, our psychophysical studies revealed that these afferents – which are very sensitive to slow, caress‐like stroking of the skin and subserve affective touch – appear to be absent or at least greatly reduced (Macefield et al., 2014). This fits with the reduced density of unmyelinated axons in cutaneous nerves in HSAN III (Aguayo et al., 1971; Pearson et al., 1971; Winkelmann et al., 1966), although skin sympathetic nerve activity – which controls the sweat glands, blood vessels and hairs – appears to be normal (Macefield et al., 2013b). So, in HSAN III we have an apparently normal density of myelinated cutaneous afferents and unmyelinated cutaneous efferents (i.e., post‐ganglionic sympathetic axons), but a reduced density of unmyelinated cutaneous sensory axons – the latter accounting for the greatly elevated temperature and cutaneous pain thresholds seen in this condition (Macefield et al., 2011; Norcliffe‐Kaufmann et al., 2017; Pearson et al., 1971).

### 1.3 Proprioception is compromised in HSAN III

**FIGURE 3:**
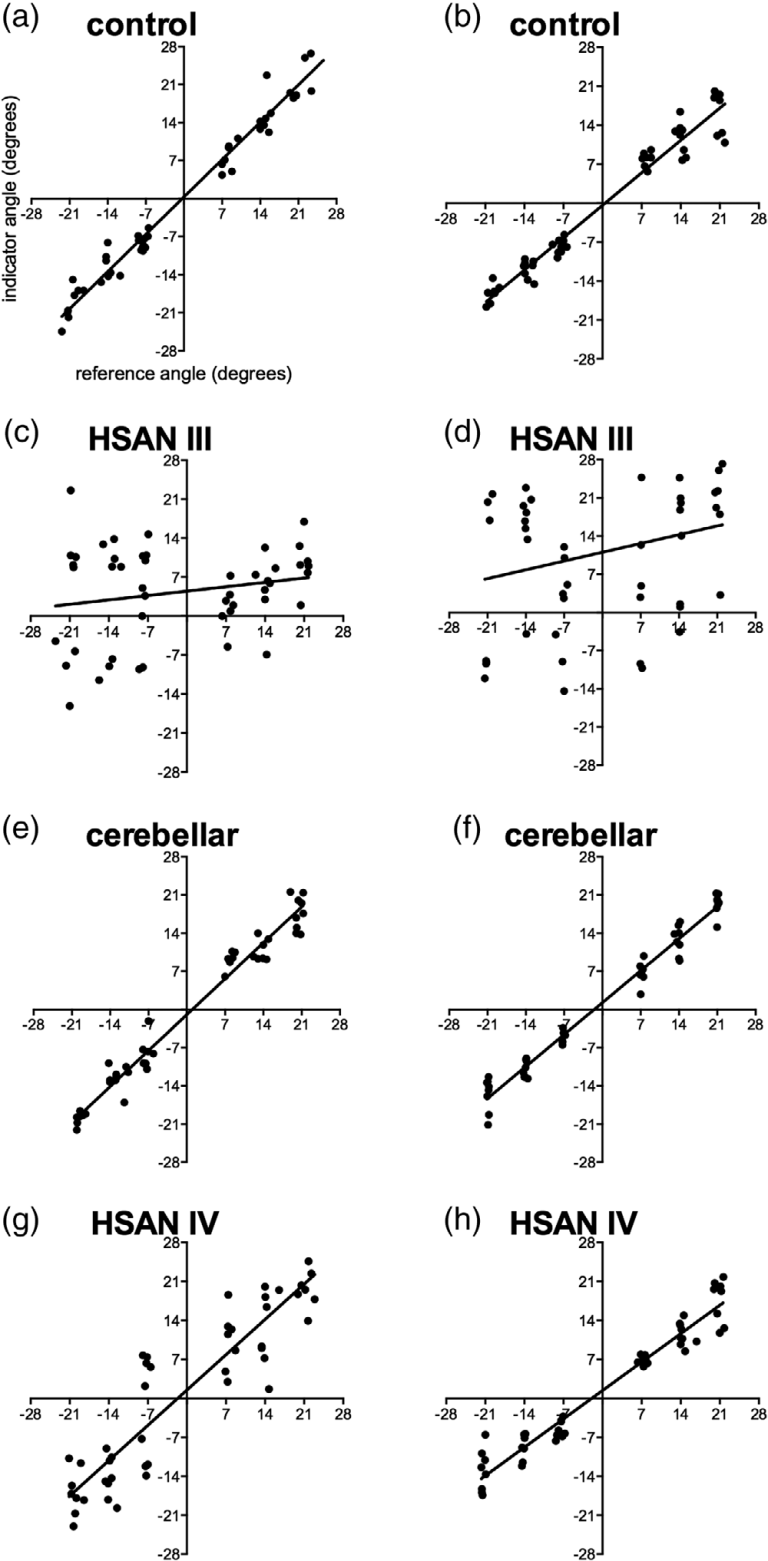
Proprioceptive acuity, assessed by passive joint angle matching at the knee in two control subjects (a, b), two patients with HSAN III (c, d), two patients with cerebellar ataxia (e, f) and two patients with HSAN IV (g, h). The angle of the reference knee is shown on the abscissa and the angle of the indicator knee is shown on the ordinate; all numbers are in degrees. Positive angles (relative the knee at 90^o^) indicate a dorsiflexed position of the knee and negative angles indicate a plantarflexed position. Lines of best fit (linear correlations) are shown superimposed on the data. Note that the slope of the relationship between the reference and indicator angles is lower than control values for the two patients with HSAN III but not those with HSAN IV or cerebellar ataxia. Reproduced, with permission, from Macefield et al. (2013a).

As noted above, given the important role muscle spindles play in encoding joint position (Proske & Gandevia, 2009, 2012), one would expect that proprioception would be compromised in HSAN III. Indeed, it has long been known that this is the case: clinical assessment of proprioception, typically performed by the neurologist moving the big toe up or down and asking the patient to report the direction of movement, is greatly affected in HSAN III. Moreover, patients perform poorly on the finger‐to‐nose pointing task, which also supports a sensory disturbance. We conducted a systematic investigation of proprioception at the knee joint, using passive joint‐angle matching – one leg is rotated into flexion or extension and the participant has to identify when the second leg is passively moved into the same angle, all without vision or extraneous cues. Naturally, healthy participants perform very well, whereas patients with HSAN III perform poorly (Macefield et al., 2013a), as shown in Figure 3a‐d. By comparison, patients with cerebellar ataxia also perform well (Fig. 3e,f); theirs is not a sensory disturbance. Interestingly, the range of matching errors was wide in HSAN III: while most had larger errors than controls, some had errors within the normal range. Moreover, matching error was positively correlated to the degree of ataxia, as assessed using the Brief Ataxia Rating Score (Scmahmann et al., 2009), in the HSAN III patients but not in patients with cerebellar ataxia (Macefield et al., 2013a). Another type of hereditary sensory and autonomic neuropathy – type IV – does not exhibit ataxia, does have functional muscle spindles (Macefield et al., 2011) and has normal proprioception (Figure 3g,h).

**FIGURE 4:**
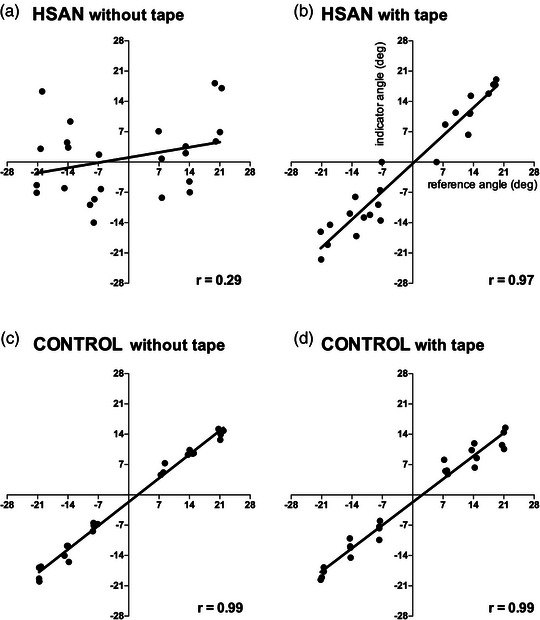
Correlation between reference and indicator angles for one HSAN III patient before (a) and after (b) taping of the knee joints, and an age‐matched control subject before (c) and after (d) taping. Angles of the reference knee are shown on the *x*‐axis, angles of the indicator knee on the *y*‐axis. Extension of the knee joint is shown as positive values, flexion as negative values. Taping increased the gradient (slope) of the relationship between the reference and indicator knee joint angles and decreased the scatter of the matched joint angles, representing an improvement in proprioceptive accuracy. Correlation values are also indicated in each condition. Reproduced, with permission, from Macefield et al. (2016).

Building on the idea that large‐diameter cutaneous inputs contribute to proprioception, and that despite the loss of muscle spindle afferents myelinated cutaneous afferents are present in HSAN III, we thought that those patients with normal matching errors at the knee joint were able to rely on proprioceptive signals coming from the skin. To test this, we applied longitudinal strips of elastic tape across the anterior and posterior aspects of the knee joint to increase tensile strain in the skin and hence increase stretch‐related activity in tactile afferents. Sure enough, this improved proprioception enormously, bringing matching errors from those patients with the worst proprioceptive acuity back to within normal limits (Figure 4); evidently, the augmented tactile input made up for the loss of muscle spindles (Macefield et al., 2016). Curiously, proprioception was normal at the elbow, and taping had no effect (Smith et al., 2020). If we assume that muscle spindles are absent throughout the body, including in the muscles that act on the elbow joint, the most parsimonious explanation is that cutaneous afferents were providing the proprioceptive information at the elbow. Indeed, we know that applying longitudinal skin stretch on one side of the joint and compression on the other, emulating changes in how the skin behaves during joint rotation, can induce illusions of joint rotation in the absence of actual rotation (Collins et al., 2005); it is likely the slowly adapting type II and III afferents in hairy skin that are responsible for this (Edin, 2001, 2004; Edin & Johansson, 1995).

### 1.4 Manual motor performance is compromised in HSAN III

The Purdue Pegboard task (Lafayette Instrument Co., Lafayette, IN, USA) is a timed test of manual dexterity that requires participants to use a precision grip between thumb and index finger to pick up a small metal cylinder (diameter 2.5 mm, length 25 mm) and insert it into a hole of the same diameter into the pegboard – rows of 25 holes, each separated by 10 mm. The test has been used clinically to demonstrate reductions in manual dexterity in, for example, carpal tunnel syndrome (Amirjani et al., 2011) and Parkinson's disease (Růžička et al., 2016). The patients with HSAN III were rather clumsy; there was a noticeable difficulty in picking up the objects as well as difficulty in inserting them into the pegboard holes, and scores were much lower than for the controls. However, applying elastic tape to the anterior and posterior aspects of the hands and wrist, the elbow and on the lateral aspect of the shoulder did not improve dexterity (Smith et al., 2018). This tells us that while cutaneous afferents can contribute significantly to proprioception, increasing cutaneous input cannot make up for the lack of muscle spindles in performing the precision sensorimotor task studied here.

## 2 DISCUSSION

Although muscle strength is not affected in HSAN III, motor function is clearly affected: patients are ataxic in all limbs and fine motor control is greatly compromised. Our use of intraneural microelectrodes to record from peripheral nerves in these patients revealed that the large myelinated muscle spindle afferents are absent in HSAN III, but that the large myelinated cutaneous afferents are present. Although we have only studied the lower leg and the hand, it is highly likely that functional muscle spindles are absent throughout the body, which would fit with the embryological model of Hamburger and Levi‐Montalcini (1949): muscle spindle afferents fail to develop because of the failed second wave of migration of neural crest cells from the dorsal root ganglion; this failure also accounts for the loss of temperature and pain sensitivity in the skin. By contrast, cutaneous afferents are established from the first wave of migration, which accounts for the preservation of cutaneous mechanoreceptors and the apparently normal tactile sensibility in HSAN III. Mice with a genetic mutation that results in a deficiency in transcription factor EGR3, responsible for the formation of muscle spindles, walk with a waddling, uncoordinated gait; they also exhibit abnormal positioning of the limbs, suggestive of impaired proprioception, and scoliosis (Tourtelotte & Milbrant, 1998). The latter is a common feature in HSAN III (Riley et al., 1954; Yoslow et al., 1971), and suggests that muscle spindles in the axial musculature are also absent in HSAN III. Another mouse model in which muscle spindles fail to form – produced by a deletion in the cytoplasmic dynein heavy chain 1 gene (*Dync1h1*) – also exhibits an unsteady jerky and wobbling gait and splayed hindlimbs and has a pure proprioceptive sensory neuropathy without any motor involvement (Chen et al., 2007). A more recent mouse model, in which ELP‐1 protein is reduced, also shows absent H‐reflexes and loss of muscle spindle afferent neurones in the dorsal root ganglia, recapitulating many of the features of HSAN III (Dietrich et al., 2012; Morini et al., 2016). The major mutation leads to variable tissue‐specific skipping of exon 20 in the *IKBKAP* transcript and hence reduced levels of IKAP/ELP‐1 protein, with the lowest levels of expression being in the central and peripheral nervous system (Cuajungco et al., 2003). Moreover, as in humans with HSAN III, gait ataxia, postural disturbances and spinal deficits, including kyphoscoliosis, worsen progressively in postnatal life (Dietrich et al., 2012; Morini et al., 2016). Importantly, correction of this splicing deficit rescues the phenotype (Morini et al., 2019).

## 3 CONCLUSIONS

Individuals with HSAN III have a complex phenotype that includes marked ataxia. By undertaking microelectrode recordings from the common peroneal and ulnar nerves we have shown that functional muscle spindles are absent in HSAN III, although large‐diameter cutaneous afferents are present and appear physiologically normal. The absence of muscle spindles may explain the poor proprioception at the knee joint, as assessed by passive joint‐angle matching. However, curiously, proprioception was normal at the elbow. Despite this, reaching and lifting small objects using the precision grip was greatly compromised, arguing for an important role of muscle spindles in this sensorimotor task. Interestingly, unlike those rare cases of complete large‐fibre sensory neuropathy, in which large‐diameter cutaneous as well as muscle afferents are lost but small‐diameter afferents are preserved (Cole & Sedgwick, 1992; Lajoi et al., 1996), our patients do have access to sensory input from the skin. Increasing cutaneous feedback improved proprioception at the knee but not at the elbow, and did not improve performance in a precision manual sensorimotor task. HSAN III provides a natural model in which to study proprioception and sensorimotor performance in humans: a genetic mutation that selectively abolishes the sensory feedback provided by muscle spindles yet not that provided by cutaneous mechanoreceptors.

## AUTHOR CONTRIBUTIONS

Vaughan G. Macefield conceived the study, contributed to data acquisition, analysis and wrote the manuscript, contributed to data acquisition and analysis. Lyndon J. Smith and Lucy Norcliffe‐Kaufmann contributed to data acquisition and approved the final manuscript; Jose‐Alberto Palma and Horacio Kaufmann were involved in performing neurological assessments of the patients. All authors have read and approved the final version of this manuscript and agree to be accountable for all aspects of the work in ensuring that questions related to the accuracy or integrity of any part of the work are appropriately investigated and resolved. All persons designated as authors qualify for authorship, and all those who qualify for authorship are listed.

## CONFLICT OF INTEREST

All authors declare they hold no competing interests.
